# Investigating Beta-Variational Convolutional Autoencoders for the Unsupervised Classification of Chest Pneumonia

**DOI:** 10.3390/diagnostics13132199

**Published:** 2023-06-28

**Authors:** Serag Mohamed Akila, Elbrus Imanov, Khaled Almezhghwi

**Affiliations:** 1 Department of Biomedical Engineering, Near East University, Mersin 10, 99138 Nicosia, Turkey; 2 Department of Computer Engineering, Near East University, Mersin 10, 99138 Nicosia, Turkey; elbrus.imanov@neu.edu.tr; 3 Electrical and Electronics Engineering, College of Electronics Technology Tripoli, Tripoli 00000, Libya; khaldalmezghwi84@gmail.com

**Keywords:** chest pneumonia, classification, unsupervised learning, convolution, autoencoder, variational

## Abstract

The world’s population is increasing and so is the challenge on existing healthcare infrastructure to cope with the growing demand in medical diagnosis and evaluation. Although human experts are primarily tasked with the diagnosis of different medical conditions, artificial intelligence (AI)-assisted diagnoses have become considerably useful in recent times. One of the critical lung infections, which requires early diagnosis and subsequent treatment to reduce the mortality rate, is pneumonia. There are different methods for obtaining a pneumonia diagnosis; however, the adoption of chest X-rays is popular since it is non-invasive. The AI systems for a pneumonia diagnosis using chest X-rays are often built on supervised machine-learning (ML) models, which require labeled datasets for development. However, collecting labeled datasets is sometimes infeasible due to constraints such as human resources, cost, and time. As such, the problem that we address in this paper is the unsupervised classification of pneumonia using unsupervised ML models including the beta-variational convolutional autoencoder (β-VCAE) and other variants, such as convolutional autoencoders (CAE), denoising convolutional autoencoders (DCAE), and sparse convolutional autoencoders (SCAE). Namely, the pneumonia classification problem is cast into an anomaly detection to develop the aforementioned ML models. The experimental results show that pneumonia can be diagnosed with high recall, precision, *f*_1_-score, and *f*_2_-score using the proposed unsupervised models. In addition, we observe that the proposed models are competitive with the state-of-the-art models, which are trained on a labeled dataset.

## 1. Introduction

The lung is a very crucial organ of the human body. It is situated in the thorax region and primarily responsible for respiration [1]. All the cells in the body require oxygen to carry out various activities. Humans typically have two lungs, one on the left and the other on the right. They continuously provide the body with the oxygen that it requires for performing different important biochemical processes [2]. The results of these biochemical processes often include carbon dioxide (CO_2_) as a by-product [3]. The human body is not conditioned for accumulating CO_2_; such an accumulation in the body is harmful. Interestingly, the lungs are also tasked with extracting the produced CO_2_ from the body [3]. The alveoli and capillaries are the specific sites where the air exchange occurs. 

The right lung is slightly larger than the left lung since the heart is also situated in the left section of the chest. Precisely, the right lung occupies 56% of the total lung volume [4]. Furthermore, the lung is involved in other functions such as the absorption and removal of water, alcohol, nitrous oxide, and drugs [5]. As such, a healthy lung is indispensable for the proper functioning of the human body. Unfortunately, there are different diseases that can plague the lungs. Some of these diseases include asthma, bronchitis, tuberculosis (TB), chronic obstructive pulmonary disease (COPD), and pneumonia [6]. Different causes including bacteria, viruses, fungi, and cigarettes may be responsible for the aforementioned diseases.

Pneumonia is an infection of the lungs involving the bronchioles and alveoli [7]. Specifically, pneumonia causes the inflammation of the alveoli. Subsequently, the alveoli collect pus and fluid so that respiration becomes problematic. Pneumonia is contagious, and, therefore, can be transmitted via airborne droplets when an infected individual coughs or sneezes [7]. Popular bacteria that cause pneumonia are Mycoplasma pneumoniae, Legionella pneumophila and Hemophilus influenzae [8]. Common viruses that cause pneumonia are influenza (flu), respiratory syncytial virus, rhinoviruses, and a coronavirus infection [9]. Popular symptoms of pneumonia include coughing, fever, shortness of breath at (near) resting state and chest pain. Considering the importance of the lung, an accurate and fast diagnosis of pneumonia has become very crucial. A wrong or late diagnosis can result in a high mortality rate [10] or expensive and long treatment procedures.

Traditionally, the diagnosis of pneumonia involves a medical specialist who evaluates the chest X-ray, CT scan, blood sample, or sputum sample of a patient [11]. A chest X-ray is perhaps the most-used method for pneumonia diagnosis. This observation may be related to the fact that X-ray acquisition is non-invasive, easy, relatively non-expensive, and fast. Consequently, it will be our focus for pneumonia diagnosis in the paper. The recent related works, along with summarized descriptions of the recent approaches for classifying chest X-rays for pneumonia, which can be found in the literature, are presented in Table 1. Like many other medical conditions [12,13,14], the assisted diagnosis of pneumonia via artificial intelligence (AI) systems can improve the accuracy of diagnosis. However, these machine-learning (ML)-based classification systems, which perform well, are often supervised models that require labeled datasets for training. One drawback, which the earlier works in Table 1 share, is the reliance on labeled datasets for learning. The work in [15] relied on the knowledge transfer from state-of-the-art deep neural networks (DNNs) for the supervised classification of pneumonia using X-rays. The knowledge from pre-trained DNNs such as AlexNet, DenseNet121, Inceptionv3, ResNet18, and GoogleNet was used as the base features for learning. The transferred layers were frozen, and new classification layers were stacked at the end of the aforementioned DNNs. Subsequently, the proposed models were fine-tuned for classifying data as ‘no pneumonia’ or ‘pneumonia’.

In [16], pre-trained DNNs including AlexNet, ResNet18, DenseNet201, SqueezeNet were used for the knowledge transfer. However, the problem was formulated as a multiclass problem, where chest X-rays were classified as having ‘no pneumonia’, ‘bacteria pneumonia’, or ‘viral pneumonia’. Another work [17] proposed the supervised classification of chest X-rays using a CNN and dynamic capsule routing units; competitive results were reported in the work. The work in [19] proposed training a CNN for feature extraction and, then, using different classifiers such as SVM, k-NN and RF to improve classification accuracy. Several experimental results were reported in the work for validating the claim. However, all the works mentioned above use labeled datasets for learning. Unfortunately, the collection of labeled datasets is not always feasible due to reasons such as cost, time, and human resources. Given the problems mentioned above, this paper proposes the unsupervised classification of pneumonia using chest X-rays. The classification task is reformulated as an anomaly detection task using different types of autoencoder models. The results of the models are especially compared against other unsupervised learning models and certain supervised models.

As such, the major expositions in this paper are the following:

(i) (Investigate unsupervised ML models such as the beta-variational convolutional autoencoder (β-VCAE) [21] and other variants, such as the plain convolutional autoencoder (PCAE) [22], denoising convolutional autoencoder (DCAE) [22], sparse convolutional autoencoder (SCAE) [23] for pneumonia diagnosis.
(ii) Study how the choice of the class that the DNN is trained on impacts the generalization performance of the aforementioned models.
(iii) Provide comparative analysis with state-of-the-art supervised models for pneumonia diagnosis. Our evaluation metrics include recall, precision, *f*_1_-score, and *f*_2_-score, which particularly takes into account false negatives and false positives.

In the remaining parts of this paper, there are the following sections: Section 2 discusses the background and problem statement. In Section 3, the proposed unsupervised ML models for the diagnosis of pneumonia are presented. In Section 4, the results of experiments from the different models investigated are given and discussed, including comparison with some state-of-the-art supervised ML models. We conclude the paper in Section 5 by summarizing the main findings from the experimental results.

## 2. Background and Problem Statements

### 2.1. Background

Given a chest X-ray, the traditional approach for pneumonia diagnosis requires a trained medical specialist to examine and evaluate the X-ray to determine if the patient has pneumonia or not. However, such a procedure can sometimes result in erroneous diagnosis due to human fatigue or random attention loss. Moreover, the time for diagnosis can quickly increase when only a few medical specialists are available to evaluate many X-ray scans. As an alternative, an AI-based computer program can be developed to aid diagnosis. Such a computer program processes the chest X-ray and searches for patterns that may reflect the presence of pneumonia. Such a computer program that is based on AI is data-driven for knowledge acquisition. The AI program is tasked to classify X-rays as having pneumonia or not, that is, to act as a binary classifier.

**Figure 1:**
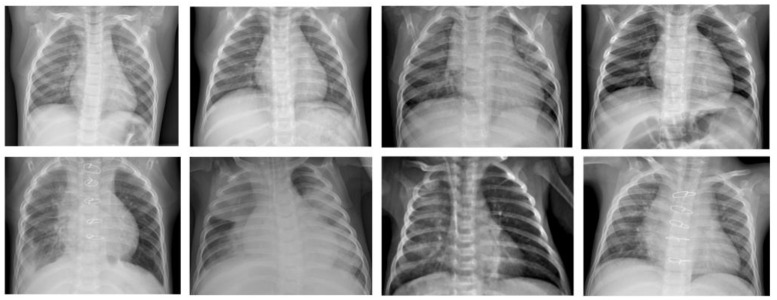
Chest X-ray samples used in this paper [24]. **Top row**: X-ray samples with no pneumonia. **Bottom row**: X-ray samples with pneumonia.

Since computer programs cannot be impacted by fatigue or distractions, they are generally reliable once properly trained for the given task. Furthermore, it is possible with AI programs to evaluate several chest X-ray records over a short period; a human examiner usually requires more time to evaluate the same number of chest X-ray records due to fatigue. The ML model that that is investigated in this work is the β-VCAE and its variants. Figure 1 shows some samples of the chest X-rays used in this work.

### 2.2. Problem Statements

Although there are existing DNN approaches for classifying chest X-rays for pneumonia, they are often supervised DNN models [25,26,27]. This means that these approaches require labeled datasets. However, collecting and labeling datasets can sometimes be very costly. At other times, we were only able to collect datasets with all samples (examples) belonging to only one class, e.g., with no pneumonia. This can occur when the disease that we are interested in diagnosing is rare so that data samples for the positive class (i.e., with pneumonia) are difficult or outrightly impossible to collect. In this scenario, most supervised DNN models such as VGG [28], ResNet [29], DenseNet [30], etc., cannot be employed. Consequently, the problem statement is the development of pneumonia classification models using unlabeled data, that is, unsupervised models.

## 3. Proposed Unsupervised Classification of Pneumonia

The objective of this work is to investigate the unsupervised classification of pneumonia using chest X-rays. What particularly distinguishes this work from the related works on the diagnosis of chest pneumonia is that the classification of pneumonia is formulated as an anomaly detection problem. That is, the developed models are trained on only samples that belong to one class and tested on samples that belong to both classes. The hypothesis is that a model that is successfully trained on only a particular class takes any sample that does not belong to the class that it was trained on as an anomalous data sample. The family of unsupervised DNNs that we explored is the CAE, which is either generative or non-generative based on the training algorithm. Generative CAEs learn the training data generation process and interpretable latent space (hidden representation), which can be sampled to generate novel data samples. In contrast, non-generative CAE do not model the training data generation process, and therefore, its latent space cannot be sampled from to generate novel samples; they simply learn a compressed representation in the latent space. In this paper, for generative CAE, we will explore the beta-variational convolutional autoencoder (β-VCAE) [21], which is a generalization of the variational convolutional autoencoder (VCAE) [31] when β = 1. In addition, we experiment with other CAEs such as the conventional CAE referred to as plain CAE (PCAE) [22], the denoising convolutional autoencoder (DCAE) [22], and the sparse convolutional autoencoder (SCAE) [23].

The developed unsupervised pneumonia classification system that is based on autoencoders can provide fast, considerably accurate, and cost-effective diagnoses without the need for a labeled dataset. This section details the proposed diagnosis system that employs different unsupervised ML models. The main stages in the classification framework are data processing and model training set up, which are discussed in the rest of the section.

### 3.1. Data and Data Processing

**Figure 2:**
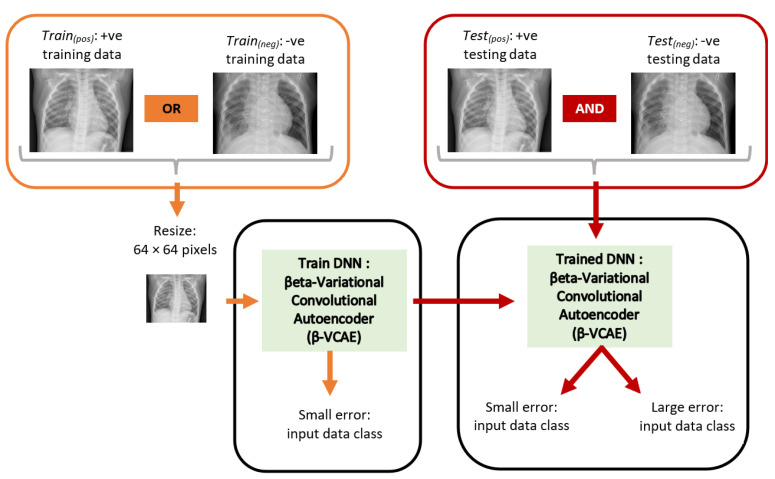
Proposed unsupervised framework for the diagnosis of pneumonia using chest X-ray. The orange items and red items show the training and testing paths, respectively. The DNN is trained using only data from Train*(pos)* or Train*(neg)*, while the model is tested using data from both Test*(pos)* and Test*(neg)*.

The chest X-ray images obtained from the database [24] have samples that reflect X-rays without pneumonia and those with pneumonia. The X-rays with pneumonia are referred to positive (+ve) samples or class, while X-rays without pneumonia are referred to as negative (−ve) samples or class. Subsequently, the positive and negative training data are abbreviated as Train*_(pos)_* and Train*_(neg)_*, respectively. Similarly, the positive and negative testing data are abbreviated as Test*_(pos)_* and Test*_(neg)_*, respectively. Figure 2 shows the overall diagnosis framework. The X-ray images have different sizes. However, the ML models employed in this paper accept input images of fixed size. As such, the X-ray images are resized to a fixed size of 64 × 64 pixels. This has the additional advantage of reducing computing hardware requirements for training and testing the ML models.

### 3.2. Proposed Unsupervised Models

The problem of pneumonia classification is formulated as an anomaly detection problem based on the autoencoder model family, where the model is trained on either only the positive class or negative. A model that is trained on the positive class is expected to have successfully learned the reconstruction of X-ray samples from the positive class, namely, giving small reconstruction errors for positive samples but high errors for negative samples during testing. Similarly, a model successfully trained on only negative samples is expected to give small reconstruction errors when tested with negative samples but a high reconstruction error when tested with positive samples. It is on this concept that the proposed models are based for diagnosing pneumonia in an unsupervised fashion.

In the paper, four different convolutional autoencoders are investigated. The motivation is to study how the formulation of the models impacts their classification performances. It is well-known that the latent space properties of autoencoders significantly contribute to their regularization characteristics and, therefore, modeling power. An autoencoder is essentially tasked to reconstruct the supplied input data in the output layer [32]. This is expected to foster the learning of interesting latent representations in the hidden layer. The hidden units learn a mapping function for reproducing the input data in the output layer. It is noteworthy that the major challenge in learning autoencoders is capturing important features in the training data, that is, learning good latent representations for the training data [33]. Although the different autoencoders used in this work employ convolution operations, we describe their operations in this section using fully connected networks (multilayer perceptrons) for the sake of simplicity. However, similar formulations can be directly extended to the autoencoders with convolution operations.

#### 3.2.1. Plain Autoencoder

**Figure 3:**
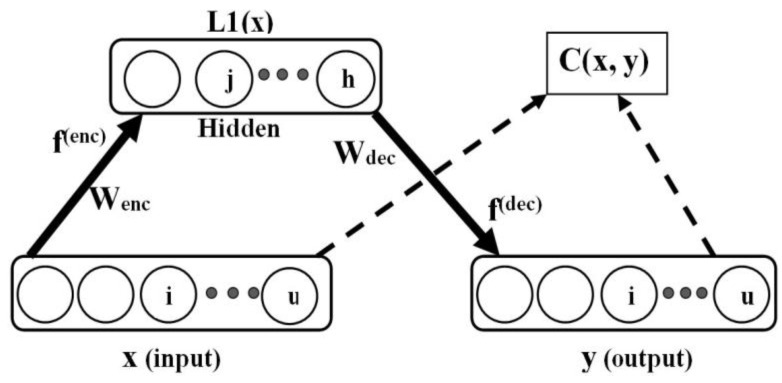
Plain autoencoder.

The plain autoencoder is shown in Figure 3, where for a supplied input pattern, x, the autoencoder is tasked with the learning of good hidden representation, L1(x), essential for the successful reconstruction of the input data in the output layer as y. Here, u is the dimensionality of the input data, and *h* is the number of hidden units. The notation, L1(x), was used to denote the first hidden layer representation (or codes), since autoencoders can be stacked to obtain a deep network (with many hidden layers). Generally, autoencoders are learned in two stages, referred to as encoding and decoding. Let the training set be of the form: $x\in R^{u}$ and $y\in R^{u}$ (as obtained in the input and output data for autoencoding) and $L1(x)\in R^{h}$.

− Encoding: the autoencoder learns the mapping of input data, x, to the hidden layer codes L1(x) as a function $f^{enc}:x\mapsto L1(x)$. This is described as given below:
  (1) $$L1(x)=g(Wx+b^{\left(L1\right)})$$
  where L1(x) is the hidden layer output, $W_{enc}^{\left(L1\right)}\in R^{h\times u}$ is the input-hidden weight matrix, $b^{\left(L1\right)}\in R^{h}$ is the bias vector for the hidden layer, and g is the activation function. The encoding phase can be conceived of as maximizing the mutual information between the input data, x, and hidden layer, L1(x) [33].
− Decoding: the decoder learns the mapping of the learned hidden layer representations (activations), L1(x), to input data, x, in the output layer as y; this is learned as a mapping function $f^{dec}:L1(x)\mapsto y$.

Note that the actual autoencoder output is *y*, while the target output is *x*, i.e., Figure 3. The decoding phase is described as follows:

(2) $$y=g(W_{dec}^{\left(L1\right)}L1(x)+b^{\left(y\right)}),$$

where *y* is the actual network output, $W_{dec}^{\left(L1\right)}\in R^{u\times h}$ is the hidden-output weight matrix, L1(x) is the hidden layer activations matrix, $b^{\left(y\right)}\in R^{u}$ is the bias vector for the output layer, and g is the activation function. The autoencoder weights, $W_{enc}^{\left(L1\right)}$ and $W_{dec}^{\left(L1\right)}$, are learned by minimizing a cost function, C(x,y;θ), for the model; where θ denotes model parameters weights and biases, that is, $\theta =\{W_{enc}^{\left(L1\right)},b^{\left(L1\right)},W_{dec}^{\left(L1\right)},b^{\left(y\right)}\}$. For real-valued inputs, the obvious choice is the mean squared error, as defined below:

(3) $$C(x,y;\theta )=arg\underset{\theta }{min}\sum_{n=1}^{N}\;\sum_{i=1}^{u}\;(x_{i}-y_{i}{)}^{2}.$$

Note that for autoencoders, the output unit denoted $y_{i}$ is tasked to reconstruct the input $x_{i}$. Furthermore, the number of output units is equal to the number of input units, u; N is the number of training patterns or samples. For binary inputs, the sum of Bernoulli cross entropies can be used as the cost function, as follows:

(4) $$C(x,y;\theta )=arg\underset{\theta }{min}\sum_{n=1}^{N}\;\sum_{i=1}^{u}\;(x_{i}log(y_{i})+(1-x_{i})log(1-y_{i})).$$

It is important to note that the autoencoder described with the convolution operations, which is used in the paper, is referred to as the plain convolutional autoencoder (PCAE).

#### 3.2.2. Denoising Autoencoder

Given sufficient model capacity (i.e., number of parameters), the plain autoencoder can undesirably learn to copy the input as the output without learning useful latent representations. As such, autoencoders can be learned with important and additional constraints that encourage the network to learn better hidden representations that are useful for subsequent discriminative tasks, e.g., improve model optimization and regularization [34]. An interesting constraint is the denoising training criterion, where input data are stochastically corrupted to some specified degree (usually 5–50%), and the autoencoder is tasked with the reconstruction of the clean (uncorrupted) data in the output layer; this model is referred to as a denoising autoencoder (DAE) [35]. The way the denoising autoencoder is learned can provide an interpretation as a generative model. Let the stochastic process where the noisy input data $\ddot{x}$ is generated from clean input data x be represented as $\kappa :x\mapsto \ddot{x}$. Then, the denoising training criterion is a way to approximate the stochastic operator $\kappa ’:\ddot{x}\mapsto x$. In this work, we implement the zero-masking noise, where input data are randomly set to zero with some pre-defined probability, σ. The DAE with the convolution operations is referred to as a convolutional autoencoder (CDAE).

#### 3.2.3. Sparse Autoencoder

An obvious concern with autoencoders is the possibility of the model to simply duplicate (without learning useful hidden representations) the input data in the output layer in view of maximizing the mutual information of input data, x, and hidden representation, L1(x) [22]. Hence, the application of sparsity can be employed as a way to constrain autoencoders to learn useful hidden representations for input data [22]. The hidden units are constrained to have a small pre-defined activation value, z [36]. From Figure 3, the computed sparsity parameter, $\overset{˘}{z}$, for a hypothetical hidden unit, j, can be obtained using

(5) $${\overset{˘}{z}}_{j}=\frac{1}{N}\sum_{n=1}^{N}\;o_{j}x_{n},$$

where $O_{j}$ is the output (or activation) of hidden unit j, $x_{n}$ is the training input pattern vector (sample) with index n, and N is the number of training samples. The main motivation behind sparsity is to constrain the hidden unit j such that $\overset{˘}{z}=z$.

An inspection of (5) reveals that such a constraint enforces the average activations of hidden unit j to have small values over training samples ${\{x}_{n}{\}}_{n=1:N}$ to realize small values of $\overset{˘}{z}$. The Kullback Leibler (KL) divergence described in (6) can be used to measure the deviation of distribution z from $\overset{˘}{z}$ [34] and, hence, optimize the whole model.

(6) $$KL(z∥{\overset{˘}{z}}_{j})=zlog\frac{z}{{\overset{˘}{z}}_{j}}+(1-z)log\frac{\left(1-z\right)}{\left(1-{\overset{˘}{z}}_{j}\right)}$$

It is important to note that $(z∥\overset{˘}{z}$) = 0 for $\overset{˘}{z}$= z. For the overall model cost minimization, the KL divergence is added to the mean squared error. Hence, the new overall cost function C(x,y;θ) is expressed in (7), whereas γ emphasizes the effect of the sparsity constraint.

(7) $$C(x,y;\theta )=arg\underset{\theta }{min}\sum_{n=1}^{N}\;\{\sum_{i=1}^{u}\;(x_{i}-y_{i}{)}^{2}+\gamma (\sum_{j=1}^{h}\;KL(z∥{\overset{˘}{z}}_{j}))\}.$$

The SAE with the convolution operations is referred to as a sparse convolutional autoencoder (SCAE).

#### 3.2.4. Beta-Variational Autoencoder

The beta-variational autoencoder (β-VAE) [21] is a generative model, and it is well-known for its structured latent space property, which makes the sampling of novel plausible samples interesting. This is an attribute that the other autoencoders lack, since their latent spaces are not structured or continuous (i.e., smooth) such that sampling from them is not guaranteed to generate semantically meaningful samples. Interestingly, the continuous latent space was argued to regularize the model even for tasks that do not require generating novel samples. Namely, the latent space of the β-VAE is constrained to be continuous manifolds so that the data reconstructed from it are more plausible. The second term in (8) is responsible for the smoothness constraint. This, in turn, is expected to improve its performance for anomaly detection and, therefore, the diagnosis of chest pneumonia.

In simple terms, this autoencoder has two terms in its cost function, as shown in the following expression:

(8) $$C(x,y;\theta )=arg\underset{\theta }{min}\sum_{n=1}^{N}\;\{\sum_{i=1}^{u}\;\left(x_{i}-y_{i}{)}^{2}+\beta \left(KL\left[q_{\varphi }\left(L1\left(x\right)|x_{i}\right)∥p\left(L1(x)|x\right)\right]\right)\right\}$$

where $q_{\varphi }\left(L1\left(x\right)|x_{i}\right)$ is the model encoder that is used to approximate the intractable $p\left(L1(x)|x\right)$, which is generally chosen to have the isotropic unit Gaussian distribution. The first term in the cost function is the typical reconstruction error, and the other term is the latent space regularization that encourages smoothness and structure. It can be noted that the β-VAE is a generalization of the conventional VAE proposed in work [31], where we can set β = 1. The β-VAE with the convolution operation is referred to as β-VCAE.

### 3.3. Model Evaluation Metrics

The evaluation metrics for the models in this work are recall, precision, *f*_1_-score, and *f*_2_-score. The chosen metrics ensure that the model evaluation is thorough. For example, class distribution impact, false positive, and false negative results are reasonably evaluated. Considering that the misdiagnosis of pneumonia can be life-threatening, the main metric for results comparison will be the *f*_2_-score. The *f_2_*-score especially highlights the false negatives predicted by the model, which are very undesirable in this application. The definitions of the evaluation metrics are as follows, where TP, FN, and FP are true positive, false negative, and false positive, respectively.

(9) $$Recall=\frac{TP}{TP+FN}$$

(10) $$Precision=\frac{TP}{TP+FP}$$

(11) $$f_{1}-score=\frac{2\times Recall\times Precision}{Recall+Precision}$$

(12) $$f_{2}-score=\frac{5\times Recall\times Precision}{Recall+4\times Precision}$$

## 4. Experiments

The dataset details and experimental settings, along with results and discussion, are presented here. The experiments were carried out using a workstation with 32 GB random access memory (RAM), intel core-i7 CPU, Nvidia GTX1080Ti GPU, which runs Windows 10 OS.

### 4.1. Dataset Preparation

There are 3875 positive data (chest X-ray) samples and 1341 negative data samples in the dataset used in this paper. The two data preparation scenarios in the work are the following.

(i) Train and validate the model on only positive data samples, that is, using only Train*_(pos)_* and Val*_(pos)_*. Test the trained models on both positive and negative data samples, that is, using Test*_(pos)_* and Test*_(neg)_*.
(ii) Train and validate the models on only negative data samples, that is, using only Train*_(neg)_* and Val*_(neg)_*. Test the trained models on both negative and positive data samples, that is, using Test*_(neg)_* and Test*_(pos)_*.

For Train*_(pos)_*, the data samples from the positive class are divided into training, validation, and testing sets in the ratio 60%:20%:20%, respectively. All the negative class data samples are used as testing data. For Train*_(neg)_*, the data samples from the negative class are partitioned into training, validation, and testing sets in the ratio 60%:20%:20%, respectively. All the data samples from the positive class are taken as taken as testing data. The details of the data preparation for model training are given in Table 2, where positive and negative classes are abbreviated as +ve and −ve, respectively. The pixels in the images are normalized to the range 0 to 1.

### 4.2. Model Architecture and Training Settings

Herein, the details of the training settings for the different models are discussed. All models have a single filter in the input and output layers that allow the reconstruction of the input images in the output layers of the different models. All the constructed models have either two or four hidden layers, which are convolution layers. As such, we can observe how the number of hidden layers impact model performance. The models with two hidden layers have 16 filters in both hidden layers. The models with four hidden layers have 32, 16, 16, and 32 filters, consecutively. All filters are of spatial size 3 × 3. The hidden layers use the rectified linear activation function, while the output layer uses the sigmoid function. The model hyperparameters are chosen by heuristics using the validation data. The initial learning rate is set at 0.1 and momentum rate at 0.9. The learning rate is decayed during model training every 3 epochs by a factor of 0.2 to facilitate model convergence to one of the good local optima. The batch size is 32, and the models are trained for 10 epochs. The threshold for class association is taken as the maximum reconstruction error on the validation data.

### 4.3. Results and Discussion

The results of the different models obtained using Train*_(pos)_* and Train*_(neg)_* are reported in this section. Furthermore, we compare the results of our model, which employ unsupervised learning with the state-of-the-art models that use supervised learning. Table 3, Table 4, Table 5 and Table 6 show the testing results for the different data preparation settings discussed in Section 4.1 (i.e., Table 2) on evaluation metrics such as recall, precision, *f*_1_-score, and *f*_2_-score. Table 3 shows the results of the models with two hidden layers trained using Train*_(pos)_* settings, that is, only positive samples as input but tested using both positive and negative samples. Table 4 reports similar experimental results with models that have four hidden layers.

It is seen in Table 3 that the β-VCAE models provides the best results based on both *f*_1_-score and *f*_2_-score, β-VCAE with β = 5, which enforces a more structured latent space that slightly outperforms β-VCAE with β = 1. The DCAE with σ = 0.1 outperforms the DCAE with σ = 0.05; we conjecture that using σ = 0.1 imposes more regularization on the model and, therefore, better performance. The SCAE model with z = 0.3 performs better than when z = 0.5.

Table 3 can be observed. First, it is seen that increasing the number of hidden layers results in some performance improvements on the models when comparing Table 3 and Table 4. Again, the β-VCAE models give the best results. Using training settings Train*_(neg)_*, Table 5 and Table 6 show the results of the models with two and four hidden layers. The models are trained on only the negative class samples but tested on both negative and positive class samples. Importantly, it is seen in the experiments that the performances of these models lag behind the corresponding models trained using Train*_(pos)_*; compare Table 3 with Table 5 and Table 4 with Table 6. Similarly, Table 5 and Table 6 show that the β-VCAE models give the best results in comparison with other models. Again, this reflects the crucial importance of the structured latent space property of β-VCAE, which the other autoencoders lack. Overall, it is seen that the β-VCAE model gives the best performance in comparison to the other autoencoders. We posit that the nature of the latent space of the β-VCAE model discussed in Section 3.2.4 can be related to its success. Namely, the structured latent space ensures that data points are constrained within a plausible manifold from which the output of the model is reconstructed. The other autoencoders do not possess this interesting attribute, as the manifold for the latent representations is not constrained. Consequently, they can generate implausible data, which reflects in lower generalization performance.

**Figure 4:**
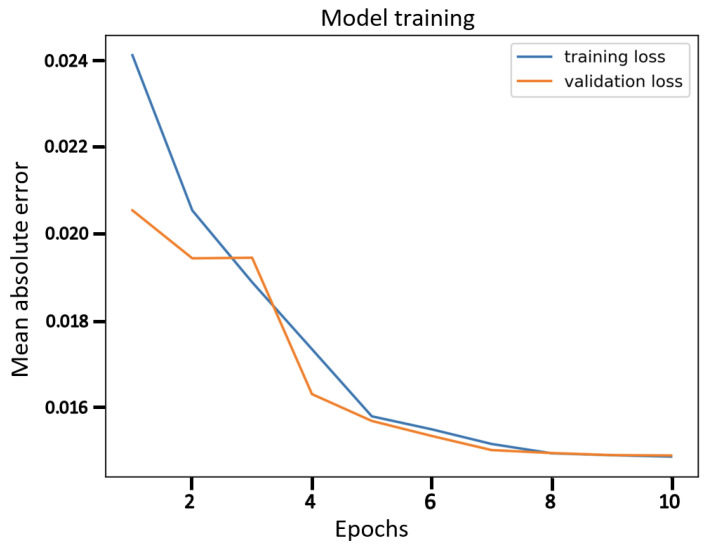
The training curve for β-VCAE with β = 5 as reported in Table 7.

**Figure 5:**
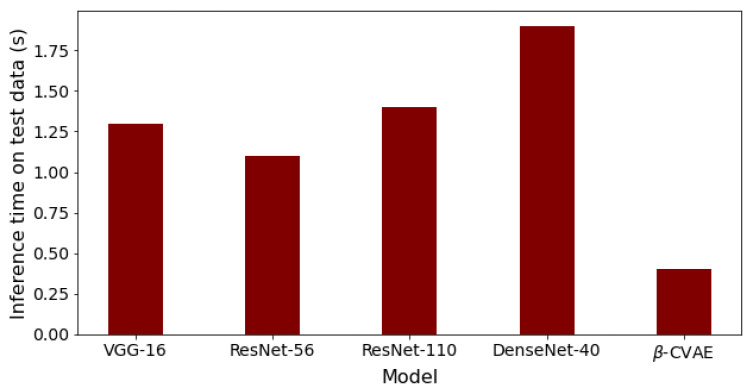
Inference times for the different models on the testing data. The results follow from the comparison results reported in Table 7.

Finally, the comparison of our best results obtained using β-VCAE with supervised models is considered. Namely, popular supervised DNNs such as the VGG-16, ResNet-56, ResNet-110, and DenseNet-40 are used for the study. The results, including the number of model parameters and depth, are shown in Table 7, where the β-VCAE models are trained using Train*_(pos)_* settings, and thus, the results are taken from Table 4. The supervised models are trained and tested by combining the same positive samples used for β-VCAE and the negative samples in Table 2. It is seen in Table 7 that the supervised models slightly outperform the proposed β-VCAE models that are unsupervised. However, when the different costs associated with collecting labeled data for training are taken into consideration, our unsupervised models are competitive and can be successfully employed for the diagnosis of pneumonia using chest X-rays. The training curve for β-VCAE with β = 5, which is reported in Table 7, is given in Figure 4. Furthermore, in comparison to the supervised models, the proposed models have significantly fewer layers and parameters, and thus, training the β-VCAE model is many times faster than the supervised models. Importantly, the inference times of the different models are especially important, since these reflect the maximum number of diagnoses that can be made within a given period. As such, the inference times of the different models are shown in Figure 5. It is observed that the proposed β-VCAE model has significantly smaller latency during testing compared to the supervised models. This is an additional advantage for the proposed model in comparison to the supervised models.

The results of the unsupervised models are quite surprisingly competitive when compared with the supervised learning models in Table 7. Our explanation is that by casting the classification problem into an anomaly detection task that autoencoders are known to perform well, the classification problem becomes easy. The autoencoder only has to learn good latent representations that partition the decoded (reconstructed) output into the two data classes, positive and negative pneumonia cases. The limitation of the proposed solution that is formulated as an anomaly detection problem in the paper is that it limits diagnosis to two classes, i.e., binary classification problems. For problems with multiple classes, the proposed solution cannot be directly applied. However, supposing that training data for all classes in a multiple classification task are available, it would be interesting to study if training an autoencoder model for each class and then ranking the errors of all the autoencoders for a given data sample during testing gives interesting results.

The major challenge in the investigation is computational resources for training the models in a reasonable time. A workstation with a GPU that has a memory of over 6 Gigabytes was found sufficient for the experiments. The training time can be further improved by using high-end GPUs that allow a larger batch size.

## 5. Conclusions

This work investigates the diagnosis of pneumonia in the scenario where the available dataset is unlabeled using a beta-variational convolutional autoencoder (β-VCAE). The work also considers the plain convolutional autoencoder (PCAE), convolutional denoising autoencoder (CDAE), and convolutional sparse autoencoder (CSAE) for the diagnosis problem. The diagnosis problem is formulated as an anomaly detection problem so that the models are trained on either only the data from the positive or negative class. The findings show that the autoencoders can be successfully applied to the problem, especially when they are trained on the positive class samples. All the models achieve an over 97% *f*_2_-score performance. We note that the β-VCAE consistently gives the best results among the different autoencoder models investigated. A comparison with supervised models such as VGG-16, ResNet-56, ResNet-110, and DenseNet-100 shows that the proposed β-VCAE, which is unsupervised, gives very competitive results and requires a significantly shorter time for testing. As such, a considerably larger number of diagnoses can be performed with the β-VCAE in comparison to the supervised models.

## Figures and Tables

**Table 1:** Related works summary for the classification of pneumonia using chest X-ray.

Study	Classification System Details
Chouhan et al., 2020 [15]	Transfer learning from AlexNet, DenseNet121, Inceptionv3, ResNet18, and GoogleNet for supervised classification.
Rahman et al., 2020 [16]	Knowledge transfer using AlexNet, DenseNet201, ResNet18, and SqueezeNet for supervised classification.
Mittal et al., 2020 [17]	Supervised classification using CNN and dynamic capsule routing.
Elshennawy et al., 2020 [18]	Used ResNet152V2, MobileNetV2, and LSTM for supervised classification.
Masad et al., 2021 [19]	Employed CNN with support vector machine (SVM), k-nearest neighbours (k-NN), and random forest (RF) for supervised classification.
Singh et al., 2020 [20]	Supervised quaternion convolutional neural network (QCNN) for end-to-end classification.

**Table 2:** Chest X-ray dataset preparation.

Data	Train_(pos)_, Val_(pos),_ Test_(pos)_ andTest_(neg)_	Train_(neg)_ Val_(neg)_ Test_(neg)_ and Test_(pos)_
Training	2325 (+ve class: 60%)	805 (-–ve class: 60%)
Validation	775 (+ve class: 20%)	268 (-–ve class: 60%)
Testing	775 (+ve class: 20%)	268 (-–ve class: 60%)
Testing	268 (-–ve class: 60%)	775 (+ve class: 20%)

**Table 3:** Testing results of DNN models with two hidden layers obtained using Train*_(pos)_*.

Model	Recall	Precision	*f*_1_-Score	*f*_2_-Score
β-VCAE (β = 1)	98.69%	91.88%	95.16%	97.25%
β-VCAE (β = 5)	**99.37%**	**92.97%**	**96.06%**	**98.02%**
PCAE	98.05%	91.25%	94.53%	96.61%
DCAE (σ = 0.1)	98.43%	91.64%	94.91%	96.99%
DCAE (σ = 0.05)	97.11%	91.24%	94.08%	95.88%
SCAE (*z* = 0.3)	98.54%	91.22%	94.73%	96.94%
SCAE (*z* = 0.5)	97.23%	91.55%	94.30%	96.04%

**Table 4:** Testing results of the DNN models with four hidden layers obtained using Train*_(pos)_*.

Model	Recall	Precision	*f*_1_-Score	*f*_2_-Score
β-VCAE (β = 1)	98.78%	93.95%	96.30%	97.77%
β-VCAE (β = 5)	**99.69%**	**94.59%**	**97.07%**	**98.63%**
PCAE	98.85%	92.66%	95.65%	97.55%
DCAE (σ = 0.1)	98.91%	93.74%	96.26%	97.83%
DCAE (σ = 0.05)	98.45%	93.36%	95.84%	97.39%
SCAE (*z* = 0.3)	98.51%	93.28%	95.82%	97.41%
SCAE (*z* = 0.5)	98.68%	93.31%	95.92%	97.56%

**Table 5:** Testing results of DNN models with two hidden layers obtained using Train*_(neg)_*.

Model	Recall	Precision	*f*_1_-Score	*f*_2_-Score
β-VCAE (β = 1)	95.08%	88.93%	91.90%	93.78%
β-VCAE (β = 5)	**95.94%**	**91.33%**	**93.58%**	**94.98%**
PCAE	94.06%	89.32%	91.63%	93.07%
DCAE (σ = 0.1)	95.37%	90.78%	93.01%	94.42%
DCAE (σ = 0.05)	95.10%	90.25%	92.61%	94.09%
SCAE (*z* = 0.3)	94.75%	90.02%	92.32%	93.76%
SCAE (*z* = 0.5)	94.28%	90.39%	92.92%	93.48%

**Table 6:** Testing results of the DNN models with four hidden layers obtained using Train*_(neg)_*.

Model	Recall	Precision	*f*_1_-Score	*f*_2_-Score
β-VCAE (β = 1)	96.72%	90.53%	93.52%	95.42%
β-VCAE (β = 5)	**97.49%**	**92.81%**	**95.09%**	**96.52%**
PCAE	95.63%	92.92%	94.26%	95.08%
DCAE (σ = 0.1)	96.54%	92.38%	94.41%	95.68%
DCAE (σ = 0.05)	96.21%	92.05%	94.08%	95.35%
SCAE (*z* = 0.3)	96.85%	92.72%	94.74%	95.99%
SCAE (*z* = 0.5)	96.88%	91.64%	94.19%	95.78%

**Table 7:** Results comparison with supervised models.

Model	Parameters	Depth	*f*_2_-Score
VGG-16 [28] (supervised)	15 M	16	98.48%
ResNet-56 [29] (supervised)	0.56 M	56	98.87%
ResNet-110 [29] (supervised)	1.7 M	110	99.22%
DenseNet-40 [30] (supervised)	1.0 M	40	**99.31%**
β-VCAE (β = 1) (proposed)	0.21 M	4	97.77%
β-VCAE (β = 5) (proposed)	0.21 M	4	98.63%

## Data Availability

The data used in the paper are publicly available for download via the weblink: https://www.kaggle.com/paultimothymooney/chest-xray-pneumonia, accessed on 20 March 2023.
